# The global macroeconomic burden of musculoskeletal disorders

**DOI:** 10.1097/JS9.0000000000003072

**Published:** 2025-07-18

**Authors:** Kaijie Qiu, Canlong Wang, Xianan Mo, Guang Yang, Lu Huang, Yan Wu, Zongyou Pan

**Affiliations:** a Department of Orthopedic Surgery, Second Affiliated Hospital, School of Medicine, Zhejiang University, Hangzhou, China; b Orthopedics Research Institute of Zhejiang University, Hangzhou City, Zhejiang Province, PR China; c Zhejiang Key Laboratory of Motor System Disease Precision Research and Therapy, Hangzhou City, Zhejiang Province, PR China; d Clinical Research Center of Motor System Disease of Zhejiang Province, PR China

**Keywords:** economic burden, Global Burden of Disease, musculoskeletal disorders, value of lost welfare

## Abstract

**Background:**

Previous literature lacks comprehensive reporting on the economic burden of musculoskeletal disorders (MSDs). Our objective was to report the macroeconomic burden of MSDs, and their subcategories, including rheumatoid arthritis (RA), osteoarthritis (OA), low back pain, neck pain, gout, and other MSDs, across 183 countries and regions in 2021.

**Methods:**

Data on overall MSDs and their subcategories in disability-adjusted life years (DALYs) were collected from the Global Burden of Disease (GBD) 2019 and 2021 database. Purchasing power parity (PPP)-adjusted gross domestic product (GDP) data were obtained from the World Bank. GDP and DALY data were combined, and the value of lost welfare (VLW) method was used to estimate macroeconomic losses. All results are presented in 2021 international dollars (PPP-adjusted).

**Results:**

In 2021, MSDs were responsible for a global VLW of $2099.84 billion, representing 1.41% of global GDP. Among MSD subcategories, low back pain had the highest VLW/GDP ratio (43%), followed by other MSDs (27%) and OA (14%). The highest economic burden was observed in high-income regions (1.74% of GDP), while the lowest was in sub-Saharan Africa (0.65% of GDP). At the national level, Cyprus and Japan experienced losses exceeding 2% of GDP. Age- and sex-specific analyses further showed that the highest burden occurred among people aged 55–59 years, with females generally bearing greater costs than males, except in gout.

**Conclusion:**

MSDs impose a substantial economic burden on the global economy, especially in high-income regions. Moreover, it is anticipated that lower- and middle-income regions will also face significant economic impacts from MSDs in the future. Among the subcategories, low back pain and other MSDs contribute the most to the overall disease burden. High-income regions should prioritize cost-effective care pathways, early intervention, and access to quality rehabilitation services, while lower- and middle-income regions need to strengthen MSDs’ prevention and invest in healthcare infrastructure to better manage the growing burden.

**HIGHLIGHTS**

- The value of lost welfare (VLW) method was used to comprehensively estimate the macroeconomic burden of musculoskeletal disorders (MSDs) across 183 countries in 2021.
- MSDs caused global economic losses of 2.1 trillion USD in 2021, equivalent to 1.41% of global GDP.
- The economic burden of MSDs was highest in high-income regions and lowest in sub-Saharan Africa, with notable national-level variations.
- Low back pain and other MSDs were the largest contributors to global MSD-related welfare losses.
- The highest burden was observed among people aged 55–59 years, with females generally bearing greater costs than males, except in gout.
- The findings highlight the need for region- and age-specific strategies, including cost-effective care pathways in high-income regions and strengthened prevention and healthcare infrastructure in lower- and middle-income regions.

## Introduction

Musculoskeletal disorders (MSDs) are a group of diseases characterized by pain and limitations in physical functioning. Over the past three decades, the proportion of disability in the global disease burden has been increasing, with MSDs being the leading cause of non-fatal disability worldwide^[1–3]^. According to 2021 data, there were approximately 1.69 billion cases of MSDs globally, resulting in 118.5 thousand deaths and 161.9 million disability-adjusted life years (DALYs)^[4]^. However, epidemiological research on MSDs has been relatively scarce, possibly due to the perception that MSDs are an inevitable byproduct of aging and are non-fatal in nature^[5,6]^. Nonetheless, given the aging population and the increasing pursuit of quality of life and longevity, the impact of MSDs on overall population health needs to be addressed.

MSDs significantly contribute to both disease burden and healthcare expenditures, particularly in high- and middle-income countries^[7,8]^. According to 2016 data, MSDs were the highest-cost disease category in healthcare expenditure in the United States^[7]^. Furthermore, with economic and population growth, it is expected that developing countries will bear an increasing share of the burden of MSDs^[9]^. Therefore, understanding the global macroeconomic trends of MSDs is crucial for the equitable allocation of limited healthcare and economic resources, serving as a baseline for relevant policies, planning, and subsequent research to alleviate the burden of MSDs and ultimately improve patient care worldwide.

However, previous research has primarily focused on the disease burden of MSDs, such as incidence, prevalence, deaths, and DALYs, without assessing their global economic impact^[10,11].^ Moreover, existing economic evaluations have typically assessed a single MSD subcategory or a limited group of countries, mainly concentrating on high-income settings and commonly studied conditions like low back pain, osteoarthritis (OA), and rheumatoid arthritis (RA)^[7,8,12–15]^. Meanwhile, conditions such as neck pain, gout, and other MSDs have been largely overlooked. To date, there remains a lack of comprehensive research that systematically assesses the global macroeconomic burden of various MSD subcategories.

The value of lost welfare (VLW) is a standardized model used to assess the economic losses caused by the current disease burden. It has gained popularity in analyzing the macroeconomic impact of diseases^[16–20]^. This model combines two key indicators: DALYs and the Value of a Statistical Life (VSL). The VSL is broadly defined as the amount an individual is willing to pay to reduce the risk of mortality^[21]^. The VLW method based on VSL estimates the economic burden of disease by quantifying the relationship between money and the risk of disability or death. Compared to other estimation methods, this approach provides a more comprehensive reflection of the economic burden of disease, as it accounts for lost income, out-of-pocket spending related to medical care, the cost associated with pain and suffering, and the intrinsic value of life^[19,22]^. Therefore, the VLW method, as a willingness to pay valuation technique, is advocated by the World Health Organization (WHO) to assess the impact of diseases on overall economic welfare^[23]^.

The aim of this study is to assess the macroeconomic burden of MSDs and their various subcategories in 183 high-, middle-, and low-income countries in the year 2021.

## Methods

### Data sources

Data supporting the findings of this study can be obtained from the corresponding author upon reasonable request.

Using the Global Burden of Disease (GBD) research database, we obtained age-specific DALYs data related to MSDs for the year 2019 and 2021^[3]^. In the GBD database, MSDs were classified into six groups: RA, OA, low back pain, neck pain, gout, and an “other” category encompassing various other MSDs such as synovial and tendon disorders, disorders of bone density and structure, etc.^[2]^. The definitions for each category are provided in Supplemental Digital Content Table S1, available at: http://links.lww.com/JS9/E755.

Life expectancy, gross domestic product (GDP), and GDP per capita data were collected from the World Development Indicators database provided by the World Bank. These data were adjusted for purchasing power parity (PPP) and reported in 2021 international dollars (USD) for each country.

Countries were grouped into seven GBD study super-regions: (1) Central Europe, Eastern Europe, and Central Asia; (2) high-income; (3) Latin America and Caribbean; (4) North Africa and Middle East; (5) South Asia; (6) Southeast Asia, East Asia, and Oceania; (7) sub-Saharan Africa^[3]^.

### Calculation of VLW

The VLW model combines VSL and DALYs to estimate the macroeconomic burden^[19–21]^. Empirical determination of VSL has been limited to a few predominantly high-income countries (HICs). To estimate VSL for all countries, the following formula was employed, which utilizes known data derived from the US: VSL_peak,i_ = VSL_peak,USA_ (GDP _i_ /GDP_USA_)^IE^, where i represents the GDP per capita of country i and IE is the income elasticity^[16–20]^. We used the 2019 value for VSL_peak,USA_, which was $9 979 014, as provided by the USDA Economic Research Service.

This formula adjusts the GDP per capita of a specific country to that of the US after adjusting for PPP. Further adjustment for willingness to pay is done using the IE parameter. In this study, the term IE refers specifically to the income elasticity of the Value per Statistical Life (IE-VSL). IE-VSL is defined as the percentage change in VSL associated with a 1% change in income. It measures how individuals’ willingness to pay for reducing mortality risk changes in response to income growth^[24]^. Economic theory and empirical studies generally support that VSL increase with income, but the rate of increase – captured by IE – is debated. The standard IE for converting between high-income regions is 0.55, while higher IEs of 1.0 and 1.5 have been used for conversions from high- to low-income settings^[17,18,20,21]^. An IE greater than 1.0 implies that reductions in mortality risk are perceived as luxury goods, meaning willingness to pay rises faster than income, which may be appropriate for extrapolating VSL to low-income settings.

For this study, an IE of 1.0 was selected to minimize assumptions about willingness to pay after adjusting for GDP per capita and purchasing power. However, additional country-specific analyses using IEs of 0.55 and 1.5 were conducted to account for local willingness-to-pay assumptions after adjusting for income. Moreover, VSL is known to vary depending on age, a factor for which further adjustment was conducted^[21]^. VSL_peak_ represents the age at which individuals in an economy are willing to pay the most to prevent death, which has been found to occur around middle age^[21]^. To estimate VSL for each individual year (VSLY), VSL_peak_ was adjusted using a quadratic function f(a), which considers willingness to pay during different years of life, where a represents age, LE represents life expectancy^[19,21]^.

**Figure:**
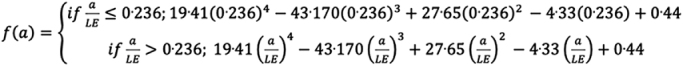

VSLYs were subsequently multiplied by age-specific DALYs and summed to obtain the final VLW in USD (2021, PPP). Furthermore, we expressed the VLW as a percentage of the GDP for the given year. Compared to the absolute VLW values, the VLW/GDP ratio highlights the relative economic pressure of a disease on a country’s economy. It serves as a relative measure that accounts for GDP differences, thereby enabling more equitable comparisons across regions or countries. It is important to emphasize that these percentages do not reflect actual GDP losses, but rather serve as a proportional representation to provide a sense of scale^[19]^.

All calculations were performed using RStudio IDE (RStudio, PBC), and the study adhered to the guidelines outlined in the Consolidated Health Economic Evaluation Reporting Standards^[25]^.

The use of generative AI in this article complies with the TITAN guideline^[26]^.

### Sensitivity analysis

Due to the uncertainties associated with the parameters used in the economic burden model, a sensitivity analysis was conducted. An IE of 0.55 was used as the upper bound, and an IE of 1.5 as the lower bound. In addition, we incorporated a scenario with an IE of 0.55 for HICs and upper-middle-income countries (UMICs), and 1.0 for lower-middle-income countries (LMICs) and low-income countries (LICs), as well as a scenario with an IE of 1.0 for HICs and UMICs and 1.5 for LMICs and LICs, to reflect differential elasticity settings across income groups. Furthermore, the lower and upper bounds of DALY estimates were also included in the model. This approach enabled us to estimate the minimum and maximum ratios of economic burden to GDP, thereby enhancing the robustness of our conclusions. The settings and results of the sensitivity analysis are provided in the Supplemental Digital Content Table S6, available at: http://links.lww.com/JS9/E755, Supplemental Digital Content Table S7, available at: http://links.lww.com/JS9/E755, Supplemental Digital Content Table S8, available at: http://links.lww.com/JS9/E755, Supplemental Digital Content Table S9, available at: http://links.lww.com/JS9/E755, Supplemental Digital Content Table S10, available at: http://links.lww.com/JS9/E755, Supplemental Digital Content Table S11, available at: http://links.lww.com/JS9/E755.

## Results

### Global level

In 2021, the global VLW due to MSDs was estimated at $2099.84 billion, accounting for 1.41% of the global GDP (These percentages indicate relative burden, not actual GDP losses). From 2019 to 2021, during the COVID-19 pandemic, the global VLW increased by $287.4 billion. However, the VLW/GDP ratio remained largely stable, with only a slight decrease of 0.008% (Supplemental Digital Content Table S2, available at: http://links.lww.com/JS9/E755).

**Figure1.**
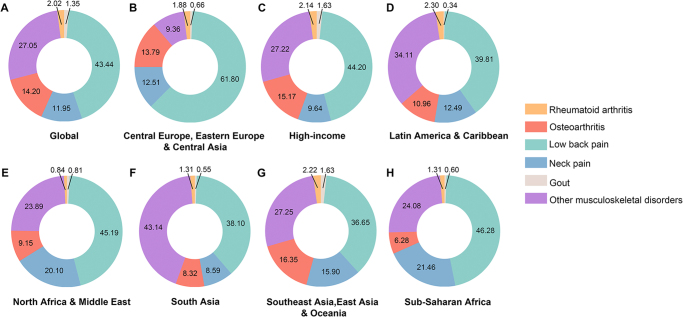
Proportion of value of lost welfare according to subcategories of musculoskeletal disorders in 2021 for global and seven super-regions.

Regarding the economic burden of MSD subcategories, low back pain contributed the largest portion to the global VLW, totaling $912.23 billion (0.61% of GDP), which accounted for 43% of the total economic burden of MSDs. Other MSDs contributed $567.92 billion (0.38% of GDP), followed by OA at $298.08 billion (0.20% of GDP). Neck pain contributed $250.90 billion (0.17% of GDP), while RA and gout contributed $42.45 billion (0.03% of GDP) and $28.25 billion (0.02% of GDP), respectively (Fig. 1 and Table 1).

**Table 1:** Value of lost welfare (VLW) and VLW/gross domestic product (GDP) attributable to musculoskeletal disorder subcategories globally and across super-regions in 2021

Super-region	RA		OA		Low back pain		Neck pain		Gout		Other MSDs	
	VLW ($ billion)	VLW/GDP (%)	VLW ($ billion)	VLW/GDP (%)	VLW ($ billion)	VLW/GDP (%)	VLW ($ billion)	VLW/GDP (%)	VLW ($ billion)	VLW/GDP (%)	VLW ($ billion)	VLW/GDP (%)
Global	42.45	0.03	298.08	0.20	912.23	0.61	250.90	0.17	28.25	0.02	567.92	0.38
Central Europe, Eastern Europe & Central Asia	3.31	0.03	24.30	0.19	108.86	0.87	22.03	0.18	1.16	0.01	16.49	0.13
High-income	23.20	0.04	164.12	0.26	478.12	0.77	104.34	0.17	17.61	0.03	294.43	0.47
Latin America & Caribbean	2.85	0.03	13.59	0.14	49.35	0.52	15.48	0.16	0.42	0.00	42.29	0.45
North Africa & Middle East	0.76	0.01	8.29	0.10	40.90	0.49	18.19	0.22	0.74	0.01	21.63	0.26
South Asia	1.90	0.01	12.10	0.09	55.42	0.40	12.50	0.09	0.80	0.01	62.75	0.45
Southeast Asia, East Asia & Oceania	10.02	0.03	73.76	0.19	165.30	0.44	71.74	0.19	7.34	0.02	122.91	0.32
Sub-Saharan Africa	0.40	0.01	1.94	0.04	14.27	0.30	6.62	0.14	0.18	0.00	7.42	0.16

**Figure2.**
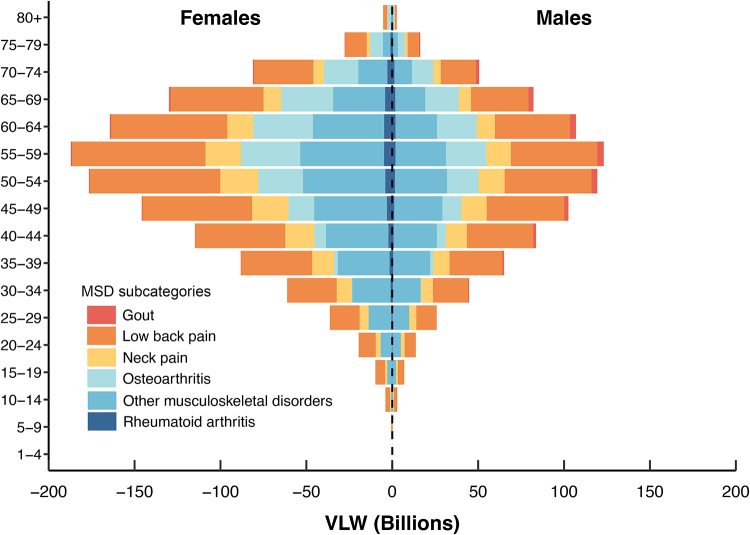
Global economic burden of musculoskeletal disorders (MSDs) in 2021, stratified by age, sex, and cause. VLW, value of lost welfare.

The age- and sex-specific analysis of the global economic burden of MSDs in 2021 reveals distinct patterns, reflecting the complex healthcare demands and cost structures across demographic groups (Fig. 2). Overall, the highest burden was observed in the 55–59 age-group, accounting for $310.19 billion (14.77% of the total MSDs burden), of which 41.20% was attributable to low back pain. When analyzed by sex, the total economic burden of MSDs was higher in females ($1252.62 billion) than in males ($847.22 billion), with females incurring greater costs across all age groups. Among the MSD subcategories, gout was the only condition for which the burden was consistently higher in males than in females across all ages (Fig. 2 and Supplemental Digital Content Table S4, available at: http://links.lww.com/JS9/E755).

### Regional level

**Figure 3.**
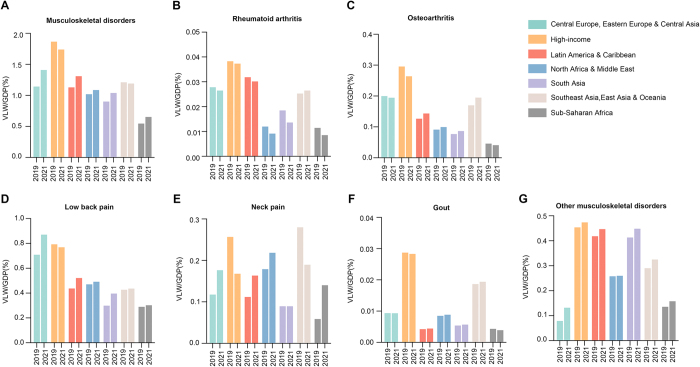
Value of lost welfare (VLW)/gross domestic product (GDP) in 2019 and 2021 by Global Burden of Disease super-region for musculoskeletal disorders overall, rheumatoid arthritis, osteoarthritis, low back pain, neck pain, gout, and other musculoskeletal disorders.

The highest VLW as a percentage of GDP attributable to MSDs was observed in the high-income super-region (1.74%, $1081.82 billion), followed by Central Europe, Eastern Europe & Central Asia (1.41%, $176.16 billion). In contrast, sub-Saharan Africa had the lowest VLW/GDP at 0.65% ($30.84 billion) (Fig. 3).

Between 2019 and 2021, the VLW/GDP decreased in the high-income super-region and the Southeast Asia, East Asia, and Oceania super-region by 0.126% and 0.019%, respectively. In contrast, all other super-regions experienced an increase in VLW/GDP, with the largest rise observed in Central Europe, Eastern Europe & Central Asia, where the increase reached 0.267% (Fig. 3 and Supplemental Digital Content Table S2, available at: http://links.lww.com/JS9/E755).

At the regional level, there were notable variations in the economic burden across different super-regions. The high-income super-region had the highest VLW/GDP for RA (0.04%, $23.20 billion), OA (0.26%, $164.12 billion), gout (0.03%, $17.61 billion), and other MSDs (0.47%, $294.43 billion). The Central Europe, Eastern Europe & Central Asia super-region exhibited the highest VLW/GDP for low back pain (0.87%, $108.86 billion), while the North Africa and Middle East super-region had the highest VLW/GDP for neck pain (0.22%, $18.19 billion) (Fig. 3 and Table 1).

Additionally, the distribution of the economic burden across subcategories of MSDs varied widely by region. Low back pain had the largest share of the VLW in the Central Europe, Eastern Europe & Central Asia super-region (61.80%), but was lowest in Southeast Asia, East Asia, and Oceania (36.65%). Other MSDs accounted for the largest share in South Asia (43.14%), while Central Europe, Eastern Europe & Central Asia had the smallest share (9.36%). For OA, Southeast Asia, East Asia, and Oceania had the highest proportion (16.35%), whereas sub-Saharan Africa had the lowest (6.28%). Neck pain had the largest share in sub-Saharan Africa (21.46%), while RA was most significant in Southeast Asia, East Asia, and Oceania (2.22%). Gout was most burdensome in the high-income super-region (1.63%) and least in Latin America and the Caribbean (0.34%) (Fig. 1).

### National level

**Figure 4.**
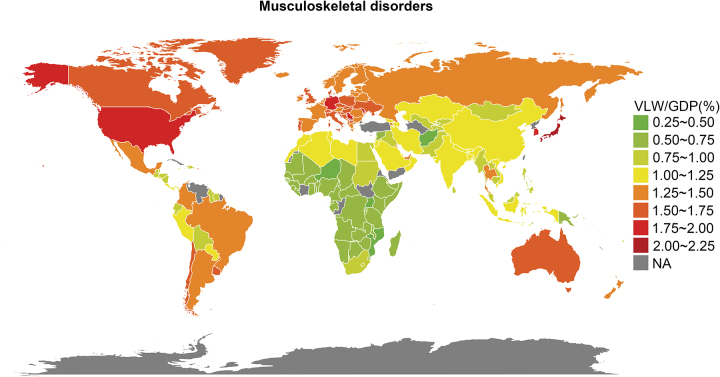
World heat maps of value of lost welfare (VLW)/gross domestic product (GDP) in 2021 by country for musculoskeletal disorders overall.

In terms of individual countries, Cyprus (VLW/GDP = 2.16%; VLW = $0.94 billion), Japan (VLW/GDP = 2.08%; VLW = $115.87 billion), and Serbia (VLW/GDP = 1.96%; VLW = $3.02 billion) had the three highest VLW/GDP. While Niger (VLW/GDP = 0.46%; VLW = $0.18 billion), Uganda (VLW/GDP = 0.47%; VLW = $0.58 billion), and Afghanistan (VLW/GDP = 0.50%; VLW = $0.43 billion) showed the lowest VLW/GDP (Fig. 4 and Supplemental Digital Content Table S5, available at: http://links.lww.com/JS9/E755).

**Figure 5.**
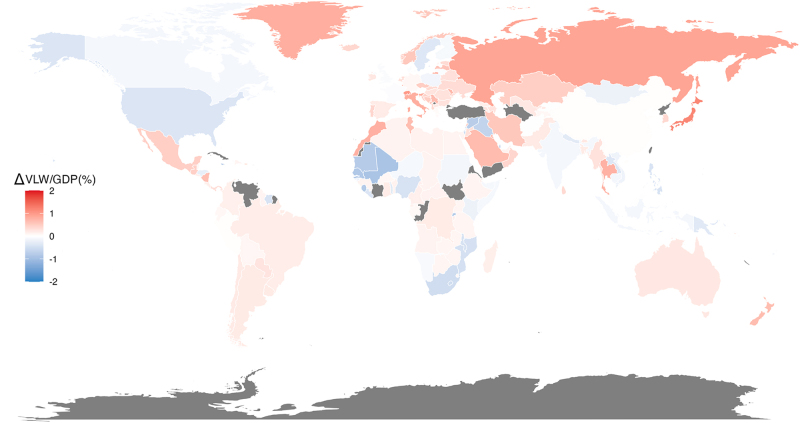
The change in value of lost welfare (VLW)/gross domestic product (GDP) caused by MSDs from 2019 to 2021 (2021 value minus 2019 value).

Between 2019 and 2021, among the 183 countries analyzed, 125 experienced an increase in the VLW/GDP due to MSDs, while 53 showed a decrease. Japan and Switzerland exhibited the largest increases, with VLW/GDP rising by 1.174% and 1.009%, respectively. In contrast, Mali and Jamaica recorded the largest declines, with VLW/GDP decreasing by 0.907% and 0.878%, respectively (Fig. 5).

At the national level, notable variations in the economic burden of MSDs were observed across countries. Finland had the highest VLW/GDP for RA (0.07%, $0.223 billion), while Japan recorded the highest VLW/GDP for OA (0.37%, $20.45 billion). Canada had the highest VLW/GDP for both gout (0.04%, $0.86 billion) and other MSDs (0.66%, $13.99 billion). Serbia exhibited the highest VLW/GDP for low back pain (1.29%, $2.00 billion), whereas Cyprus had the highest VLW/GDP for neck pain (0.31%, $0.13 billion) (Supplemental Digital Content Figure S2, available at: http://links.lww.com/JS9/E755).

Additionally, when analyzing the MSD subcategories contributing the most to the economic burden in each country, we found that low back pain was the leading cause in the majority of countries. Only six countries – Costa Rica, Ecuador, India, Mexico, Panama, and Thailand – had other MSDs as the subcategory with the highest economic burden (Supplemental Digital Content Figure S3, available at: http://links.lww.com/JS9/E755).

We listed the 2021 estimated VLW and VLW/GDP of MSDs across different super-regions under various IE values in Supplemental Digital Content Figure S1, available at: http://links.lww.com/JS9/E755 and Supplemental Digital Content Table S3, available at: http://links.lww.com/JS9/E755, and the corresponding estimates for different countries under various IE values in Supplemental Digital Content Table S8, available at: http://links.lww.com/JS9/E755, Supplemental Digital Content Table S9, available at: http://links.lww.com/JS9/E755, Supplemental Digital Content Table S10, available at: http://links.lww.com/JS9/E755, Supplemental Digital Content Table S11, available at: http://links.lww.com/JS9/E755.The trends across super-regions are stable under IE = 1.0, an IE of 1.0 for HICs and UMICs and 1.5 for LMICs and LICs, and IE = 1.5, indicating the robustness of our findings. However, when IE = 0.55 or an IE of 0.55 for HICs and UMICs and 1.0 for LMICs and LICs, the South Asia super-region and the Latin America & Caribbean super-region showed abnormally elevated VLW/GDP values (Supplemental Digital Content Figure S1, available at: http://links.lww.com/JS9/E755 and Supplemental Digital Content Table S1, available at: http://links.lww.com/JS9/E755).

## Discussion

### Key findings

Our study reveals that MSDs imposed a substantial global economic burden in 2021, with VLW estimated at 2.1 trillion USD, or 1.41% of global GDP – comparable to the economic impact of stroke^[20]^. Low back pain was the largest contributor, accounting for 43% of total MSD-related losses. The burden varied across regions, ranging from 1.74% of GDP in high-income areas to 0.65% in sub-Saharan Africa. At the national level, Cyprus and Japan experienced losses exceeding 2% of GDP. Age- and sex-specific analyses further showed that the highest burden occurred among people aged 55–59 years, with females generally bearing greater costs than males, except in gout.

Previous studies have primarily focused on the disease burden of MSDs, such as incidence, prevalence, mortality, and DALYs, while research on the economic burden of MSDs remains limited^[10,11].^ Existing economic evaluations have typically addressed individual MSD subcategories or focused on a small number of countries, with most analyses concentrated in high-income settings^[7,8,12–15]^.

Although the estimated economic burden reported in this study is not directly comparable to previous analyses of MSDs–related costs due to differences in data sources and methodologies, some general comparisons can still be made. For example, one study synthesized data from a wide range of sources, including government budgets, insurance claims, facility records, household surveys, and official statistics from the United States, using the Disease Expenditure Project methodology to estimate healthcare spending^[7]^. That analysis systematically evaluated the economic burden of 154 health conditions in the United States from 1996 to 2016 and identified MSDs as the highest-cost health category in 2016, with estimated expenditures of $380.9 billion. This is broadly comparable to our estimated U.S. MSDs burden of $436.3 billion in 2021, lending support to the plausibility and robustness of our findings. However, it is worth noting that their approach primarily focused on direct healthcare costs derived through statistical modeling and did not account for indirect costs such as productivity loss. Furthermore, their estimates are limited to the U.S. setting and are based on data that may not fully reflect more recent trends in health spending.

### Regional differences

Our study reveals that the high-income super-region exhibits the highest VLW/GDP ratio in terms of the overall economic burden of MSDs, while the sub-Saharan Africa super-region has the lowest ratio. Regional disparities in life expectancy and population structure likely explain these differences. Countries with longer life expectancy are concentrated in the high-income super-regions, while the sub-Saharan Africa super-region has the lowest life expectancy^[27]^. Additionally, in 2021, the proportion of the population aged 65 and above was much higher in the high-income super-region (18.9%) compared to the sub-Saharan Africa super-region (3.0%)^[28,29]^, which aligns with the difference in life expectancy. Longer life expectancy and population aging not only contribute to higher MSDs prevalence but also lead to a higher VSL – that is, the amount individuals or societies are willing to pay to reduce the risk of mortality. Since VSL is positively correlated with income, life expectancy, and perceived quality of life, individuals in high-income regions tend to assign greater value to each year of life saved or extended^[24]^. Therefore, under the VLW methodology, the same health loss translates into a larger economic burden in wealthier and older populations. The heightened demand for healthcare and social security services among aging populations, including treatment and rehabilitation for MSDs and long-term post-illness care, further strains healthcare systems and public finances in these regions.

Previous researches has shown that between 1990 and 2019, life expectancy experienced the fastest growth in eastern sub-Saharan Africa^[27]^. Therefore, although the current economic burden of MSDs in low- and middle-income regions dominated by the sub-Saharan Africa super-region is relatively low, it is expected to rapidly increase with the extension of life expectancy and potential aging^[30]^. The relatively inadequate healthcare facilities and services in low- and middle-income regions further contribute to the growing economic burden of MSDs^[31]^. This changing trend is evident from the growth of 20% in the VLW/GDP ratio of the sub-Saharan Africa super-region from 2019 to 2021.

### Subcategory-specific burdens

The economic burden of MSDs, as measured by VLW/GDP, varied by cause. Across the world and in most super-regions, low back pain had the highest proportion of VLW/GDP, while gout had the lowest, aligning with their respective incidence and prevalence distributions^[10,11]^. Besides imposing direct healthcare costs, low back pain significantly impacts daily functioning and work productivity, leading to substantial indirect economic burdens^[32,33]^. In many regions, the indirect economic losses caused by low back pain exceed the direct economic losses^[33]^. Implementing prevention-focused interventions in areas with a heavy economic burden from low back pain was recommended, such as improving workplace ergonomics, encouraging regular exercise, and promoting early intervention^[34]^. Additionally, expanding the coverage of affordable healthcare and rehabilitation services, along with raising public health awareness through public health campaigns, can effectively reduce both direct and indirect economic burdens^[35]^.

It is worth noting that other MSDs constituted a significant proportion in many super-regions. Specifically, in the South Asia super-region, it represents the largest proportion among all MSDs subcategories. Other MSDs encompass a heterogeneous group of MSDs, such as osteoporosis, systemic lupus erythematosus, muscle and joint infections, and sports-related injuries. Among these, osteoporosis stands out as a particularly important condition due to the substantial economic burden associated with osteoporotic fractures worldwide^[36]^. According to previous studies, osteoporosis affects approximately 10–30% of women over the age of 40 and up to 10% of men in the Asia-Pacific region^[37]^. In the South Asia super-region, India serves as a representative country where the burden of osteoporosis is of particular concern, with approximately 20% of women affected by the condition^[38]^. Notably, around 80% of the urban population in India is vitamin D deficient, and the average age at which hip fractures occur due to osteoporosis is nearly a decade earlier than in Western countries^[39]^. This high prevalence of vitamin D deficiency in India may be attributed to multiple factors, including inadequate sun exposure, low dietary intake of vitamin D, high skin pigmentation, environmental pollution, and traditional clothing practices that limit skin exposure to sunlight^[40]^.

Additionally, with the increasing number of individuals participating in sports and physical activities, the incidence of sports-related injuries and their associated economic burden is expected to rise. However, due to the lack of high-quality population-level information and limited communication between the fields of sports medicine and public health professionals and institutions, sports-related injury diseases may still be overlooked in the public health agenda^[41]^.

Thus, it is necessary to further subtype the diseases within the other MSDs and conduct high-quality data collection.

### Recommended interventions

Like other chronic noncommunicable diseases, early diagnosis and proactive preventive measures can significantly reduce the global economic burden of MSDs^[42]^. Recommended strategies include maintaining a healthy diet, engaging in regular physical activity, and adopting ergonomic interventions^[43]^. During treatment, adopting evidence-based clinical approaches, including appropriate surgical interventions where indicated, and avoiding unnecessary medication is essential, as this helps prevent patient harm, improves functional outcomes, and conserves limited healthcare resources^[34]^. Besides, effective and comprehensive rehabilitation measures can prevent the worsening of MSDs, the occurrence of complications, and lifelong consequences, thus reducing the economic burden of MSDs. In 2017, the World Health Organization launched the “Rehabilitation 2030” initiative, which not only provides resources and facilities for the provision of rehabilitation services but also offers an evidence-based list of rehabilitation interventions. This enables all regions of the world, particularly those with limited resources, to better deliver MSDs’ rehabilitation services^[44]^.

In high-income regions, where populations are rapidly aging and healthcare utilization is high, the focus should be on optimizing cost-effective care pathways, promoting early intervention, and ensuring access to high-quality rehabilitation services^[34]^. In contrast, low- and middle-income regions, where healthcare systems are often fragile, should place greater emphasis on MSDs prevention and invest in strengthening healthcare infrastructure and service delivery to better cope with the growing burden^[34,45]^. Ultimately, it is crucial to involve clinicians, researchers, and policymakers in the formulation of policies related to MSDs.

### Strengths and limitations

To our knowledge, this is the first study to quantify the global macroeconomic burden of MSDs across 183 countries. Unlike prior studies focused on individual MSD subcategories or high-income settings, our analysis includes overlooked conditions (e.g., neck pain, gout) and highlights disparities across income regions. The economic burden of MSDs includes direct costs, indirect costs, and intangible costs^[8]^. The VLW method employed in this study captures all these dimensions by considering both market and non-market losses. Unlike the value of lost output method, which only accounts for productivity losses, VLW reflects the intrinsic value of health and the psychosocial burden of illness^[19]^. This makes it especially suitable for chronic conditions like MSDs, where long-term disability and quality of life are major concerns. VLW thus serves as a valuable tool for guiding policy decisions that reflect the true burden of health conditions^[19,35]^.

Our study has several limitations. First, VSL studies are based on willingness to pay for small changes in mortality risk, and the linear assumption that is consequently made to determine the VSL is likely an oversimplification^[23]^. Second, the data used in the study heavily rely on model estimates rather than direct measurements. For instance, the VSL and VSLY in many countries were estimated based on empirical data from the United States and functions derived from studies by Viscusi and Aldy^[21]^, which may not fully reflect regional realities and could introduce bias due to differences in willingness to pay or income levels. As our sensitivity analysis showed, applying lower IE values (IE = 0.55, or 0.55 for HICs/UMICs and 1.0 for LMICs/LICs) led to abnormally elevated VLW/GDP values in regions with lower income levels, such as South Asia and Latin America & the Caribbean. Third, although GBD methods and outcomes are considered reliable, the lack of high-quality epidemiological studies on MSDs in low- and middle-income countries means that the DALY data provided by GBD are primarily model-based. Fourth, the results derived from the VLW method do not directly represent actual GDP losses, preventing direct comparisons with economic burden results obtained using other calculation methods. Lastly, our study lacks timeliness as the latest available data from GBD only goes up to 2021.

### Conclusions

MSDs impose a substantial economic burden on the global economy, especially in high-income regions. Moreover, it is anticipated that lower- and middle-income regions will also face significant economic impacts from MSDs in the future. Among the subcategories, low back pain and other MSDs contribute the most to the overall disease burden. High-income regions should prioritize cost-effective care pathways, early intervention, and access to quality rehabilitation services, while lower- and middle-income regions need to strengthen MSDs prevention and invest in healthcare infrastructure to better manage the growing burden.

## Data Availability

Data used for the analyses are publicly available from the GBD 2021 (https://ghdx.healthdata.org/gbd-2021), and the World Development Indicators database provided by the World Bank (https://databank.worldbank.org/source/world-development-indicators#). If requested, the specific methodology and data used for calculating the VLW can be provided. Please contact the corresponding author for further details

## Research registration unique identifying number (UIN)

We do not involve human subjects in our study; therefore, registration is not required.
